# Association between SARS‐CoV‐2 infections during pregnancy and preterm live birth

**DOI:** 10.1111/irv.13192

**Published:** 2023-09-21

**Authors:** Sarita Mohanty, Alan T. Tita, Michael Varner, Melissa S. Stockwell, Gabriella Newes‐Adeyi, Ashley N. Battarbee, Lawrence Reichle, Tyler Morrill, Michael Daugherty, Mirella Mourad, Raul A. Silverio Francisco, Kate Woodworth, Kristina Wielgosz, Romeo Galang, Pete Maniatis, Vera Semenova, Fatimah S. Dawood

**Affiliations:** ^1^ Centers for Disease Control and Prevention Atlanta Georgia USA; ^2^ Center for Women's Reproductive Health and Department of Obstetrics and Gynecology, Division of Maternal‐Fetal Medicine University of Alabama at Birmingham Birmingham Alabama USA; ^3^ Department of Obstetrics and Gynecology University of Utah Health Sciences Center Salt Lake City Utah USA; ^4^ Division of Child and Adolescent Health, Department of Pediatrics Columbia University Irving Medical Center New York New York USA; ^5^ Department of Population and Family Health, Mailman School of Public Health Columbia University Irving Medical Center New York New York USA; ^6^ New York‐Presbyterian Hospital New York New York USA; ^7^ Abt Associates Rockville Maryland USA

**Keywords:** COVID‐19, pregnancy, premature birth, SARS‐CoV‐2

## Abstract

We examined associations between mild or asymptomatic prenatal SARS‐CoV‐2 infection and preterm live birth in a prospective cohort study. During August 2020–October 2021, pregnant persons were followed with systematic surveillance for RT‐PCR or serologically confirmed SARS‐CoV‐2 infection until pregnancy end. The association between prenatal SARS‐CoV‐2 infection and preterm birth was assessed using Cox proportional‐hazards regression. Among 954 pregnant persons with a live birth, 185 (19%) had prenatal SARS‐CoV‐2 infection and 123 (13%) had preterm birth. The adjusted hazard ratio for the association between SARS‐CoV‐2 infection and preterm birth was 1.28 (95% confidence interval 0.82–1.99, *p* = 0.28), although results did not reach statistical significance.

## 1 BACKGROUND

More than 225,000 cases of COVID‐19 in pregnant persons were reported to the Centers for Disease Control and Prevention (CDC) during January 2020–July 2022.^1^ The cumulative number of SARS‐CoV‐2 infections among pregnant persons in the United States is likely higher because some infections are asymptomatic or may not prompt clinical testing for laboratory confirmation. Although case series and surveillance studies of COVID‐19 among pregnant persons suggest that most infections are mild, some studies have reported associations between prenatal COVID‐19 and adverse pregnancy outcomes, including preterm live birth and stillbirth.^2^, ^3^ However, studies examining the effect of prenatal SARS‐CoV‐2 infection have focused on medically attended infections that may not represent the majority of infections in the community, which are mild. Cross‐sectional or retrospective study designs are subject to misclassification of infection status and recall error, while case‐series do not include comparison with pregnant persons without infection. Understanding the impact of SARS‐CoV‐2 infection on pregnancy outcomes is important for informing guidance for pregnant persons and for characterizing the overall impact of COVID‐19. To examine possible associations between prenatal SARS‐CoV‐2 infection and risk of preterm live birth, we used data from a multisite community cohort of pregnant persons who participated in prospective systematic surveillance for symptomatic and asymptomatic SARS‐CoV‐2 infections before the SARS‐CoV‐2 Omicron variants emerged.

## 2 METHODS

### 2.1 Study design and participants

During August 2020–February 2021, the Epidemiology of SARS‐CoV‐2 in Pregnancy and Infancy (ESPI) study enrolled a cohort of pregnant women at <28 weeks gestation at three US medical centers in Salt Lake City, UT; New York City, NY; and Birmingham, AL. Cohort participants were followed with active surveillance for SARS‐CoV‐2 infections through the end of their pregnancies; the follow‐up period spanned August 2020–October 2021.

Detailed eligibility criteria and data collection procedures were previously described.^4^ At enrollment, participants completed self‐administered electronic questionnaires about their medical and pregnancy histories including whether they were diagnosed with COVID‐19 with a positive laboratory test during pregnancy before study enrollment.

From enrollment through the end of pregnancy, participants were asked to self‐collect mid‐turbinate nasal swab (MTS) specimens every week during pregnancy and ship specimens on ice packs by overnight courier to a central laboratory for reverse‐transcription polymerase chain reaction (RT‐PCR) testing for SARS‐CoV‐2. Participants also collected and shipped additional swab specimens if they experienced onset of COVID‐like illness (CLI) symptoms, defined as ≥1 of measured or subjective fever, cough, shortness of breath, sore throat, diarrhea, muscle aches, chills, or change in taste or smell. In addition, participants had serum collected at enrollment, around 26–30 weeks of gestation (if enrolled at <24 weeks gestation) and at end of pregnancy; sera were tested for antibodies to SARS‐CoV‐2 as evidence of infection.

At the end of pregnancy, participants completed self‐administered questionnaires about pregnancy outcomes and COVID‐19 vaccine receipt during pregnancy. Pregnancy outcome and COVID‐19 vaccination data were also abstracted from medical records.

### 2.2 Ethical review

The study protocol was reviewed and approved by the Columbia University Irving Medical Center Institutional Review Board (IRB), which served as the single IRB for all study sites. The CDC IRB relied on the review of the Columbia University Irving Medical Center IRB.

### 2.3 Laboratory methods

Respiratory swab specimens were tested by RT‐PCR for SARS‐CoV‐2 using the Quidel Lyra SARS‐CoV‐2 Assay or the ThermoFisher Combo Kit platform with ThermoFisher probes and primers.^5^ Serum samples were tested by xMAP SARS‐CoV‐2 Multi‐Antigen IgG Assay Accessory Kit (EUA‐IVD) (Luminex Corporation, catalog # CN‐SW74‐01), which contains three SARS‐CoV‐2 viral antigens, nucleocapsid (N), spike (S1), and receptor binding domain (RBD).

### 2.4 Study definitions

The exposure of interest was SARS‐CoV‐2 infection during pregnancy defined as ≥1 of a self‐reported laboratory‐confirmed diagnosis of COVID‐19 during pregnancy before study enrollment, RT‐PCR‐confirmed infection detected by study surveillance, or serologic evidence of SARS‐CoV‐2 infection during the study. Participants were considered to have serologic evidence of SARS‐CoV‐2 infection if they had a negative serum sample followed by a positive serum sample indicating seroconversion. Serum samples were considered positive if the spike (S1) or RBD antigens were detected in samples collected before COVID‐19 vaccination or if the N antigen was detected in samples collected after COVID‐19 vaccination.

The outcome of interest was preterm live birth, which was identified by an International Classification of Disease‐10 (ICD‐10) discharge diagnosis code of O60.1 or P07 in the delivery hospitalization medical record or defined as a live birth at gestational age <37 0/7 weeks calculated from participant‐reported estimated delivery date if medical record data were not available.

### 2.5 Sample size considerations

The goal sample size of the ESPI cohort was chosen to achieve a desired precision for calculating the cumulative incidence of SARS‐CoV‐2 infection. A priori sample size calculations for assessing the effect of SARS‐CoV‐2 on preterm birth were performing for varying effect sizes to identify the minimum effect size that could be detected with the predetermined cohort sample size. Assuming a preterm birth prevalence of 10% among persons without SARS‐CoV‐2 and a cumulative incidence of SARS‐CoV‐2 infection of ≥10%, a sample size of 1182 was determined to provide sufficient statistical power (with type I error of 5% and type II error of 20%) to detect a minimum effect size of prenatal SARS‐CoV‐2 exposure of 2.0.

### 2.6 Statistical analysis

The analysis was restricted to participants who contributed to surveillance for SARS‐CoV‐2 by submitting ≥1 MTS sample during the study period. Participants were excluded if they had an induced abortion, reported being diagnosed with COVID‐19 before pregnancy, or were seropositive at enrollment without a previous diagnosis of COVID‐19 during pregnancy because of uncertainty about whether their infection occurred before or during pregnancy. Participants with pregnancy losses (miscarriages or stillbirths) were also excluded.

The association between SARS‐CoV‐2 infection during pregnancy and perinatal outcomes was assessed using Cox proportional‐hazards regression. The time scale was weeks with week 0 defined as starting on the calculated date of conception. Participants who were lost to follow‐up or withdrawn before end of pregnancy without data collection about pregnancy outcomes were included in models and censored at last surveillance respiratory specimen collection. Full‐term pregnancies exited the risk‐set after 36 weeks gestation. SARS‐CoV‐2 infection was treated as a time‐varying covariate where a participant was considered exposed from the date of laboratory confirmation of their first infection. For participant‐reported infections before study enrollment, participant‐reported date of laboratory confirmation was used to assign infection timing. For study weeks when participants did not submit weekly MTS, infection status was imputed using study serology results. Participants who were seronegative during all serum collection timepoints without RT‐PCR‐confirmed SARS‐CoV‐2 infection were assumed to be uninfected. Participants who seroconverted during the study without RT‐PCR‐confirmed SARS‐CoV‐2 infection were assumed to have been infected during the interval when they seroconverted, and their infection timing was assigned as the midpoint between the seronegative sample and 4 weeks before the first seropositive sample.

The following variables were considered for inclusion in multivariable model: study site, age group (18–24, 25–34, and 35–50 years), highest educational level (less than college, some college or higher), presence of ≥1 chronic condition, self‐reported smoking or alcohol use during pregnancy, multiple gestation pregnancy, diagnosis of gestational diabetes or gestational hypertension, and self‐report of ≥1 psychosocial stressor during pregnancy based on a 17 question series administered at the end of pregnancy. Study site and age group were chosen a priori for inclusion in all final models. Additional covariates with a *p* value <0.1 in the initial model were retained. The final model for preterm live birth included study site, age group, highest educational level, presence of ≥1 chronic condition, diagnosis of gestational diabetes, diagnosis of gestational hypertension, current smoking status, and multiple gestation pregnancy. A Poisson regression model that does not address gestational time as a potential confounder was also run as a sensitivity analysis to estimate relative risks for comparison with published estimates from other studies.

Analyses were done in SAS version 9.4.

## 3 RESULTS

During the study period, 1176 pregnant persons were enrolled and participated in surveillance for SARS‐CoV‐2 infection (Figure S1). Of these 1176 participants, 222 were excluded (three for induced abortions, 34 for diagnosis of COVID‐19 before their pregnancy, 169 for seropositivity at enrollment without COVID‐19 diagnosis during pregnancy, and 16 for pregnancy losses). Of the remaining 954 participants, 905 (95%) were followed through the end of pregnancy and 49 (5%) were lost to follow‐up or withdrew without data collection on pregnancy outcomes. Participants in this analysis contributed a median of 13 person‐weeks of observation time with weekly MTS submission and a median of 16 person‐weeks of observation time once missing weeks were imputed using serology data.

Among the 954 participants in this analysis (Table 1), the median age was 30 years (IQR 26–34), 719 (75%) had some college education or higher, and 253 (27%) had ≥1 underlying medical condition. Participants were enrolled at a mean of 19.3 gestational weeks (standard deviation: 5.9). Overall, 185/954 (19%) participants had evidence of SARS‐CoV‐2 infection during pregnancy including 38 (4%) by self‐reported COVID‐19 diagnosis during pregnancy before enrollment, 104 (11%) with RT‐PCR‐confirmed SARS‐CoV‐2 infection by study surveillance, and an additional 43 (5%) who seroconverted during the study. Of the participants with evidence of SARS‐CoV‐2 infection during pregnancy, 23/185 (12%) were infected during the first trimester, 97/185 (52%) were infected during the second trimester and the remaining 65/185 (35%) were infected in the last trimester. Of the 104 participants with RT‐PCR‐confirmed SARS‐CoV‐2 detected by study surveillance, 27 (26%) were asymptomatic. Two infections resulted in 2‐day hospitalizations for dehydration.

**TABLE 1:** Baseline characteristics of cohort participants, *N* = 954.

	Total		SARS‐CoV‐2^a^ negative		SARS‐CoV‐2 positive		*p* value^b^
	*n* = 954	%	*n* = 769	%	*n* = 185	%	
**Study site**							
New York City, NY	242	25%	170	22%	72	39%	<0.0001
Birmingham, AL	253	27%	204	27%	49	26%	
Salt Lake City, UT	459	48%	395	51%	64	35%	
**Age at enrollment**							
18–24	154	16%	119	15%	35	19%	0.2844
25–34	601	63%	483	63%	118	64%	
35–50	199	21%	167	22%	32	17%	
**Highest education level**							
Less than college	231	24%	176	23%	55	30%	0.0496
Some college or college graduate	719	75%	590	77%	129	70%	
Missing/Decline to respond	4	0%	3	0%	1	1%	
**Health insurance**							
Private insurer	543	57%	464	60%	79	43%	<0.0001
Medicaid	346	36%	246	32%	100	54%	
Military/uniformed service insurer	3	0%	2	0%	1	1%	
Other	12	1%	10	1%	2	1%	
Missing	50	5%	47	6%	3	2%	
**≥1 chronic condition** ^**c**^	253	27%	217	28%	36	19%	0.0154
**Prepregnancy body mass index**							
<18.5	16	2%	15	2%	1	1%	0.3716
18.5–25	407	43%	331	43%	76	41%	
25–30	254	27%	198	26%	56	30%	
≥30	236	25%	192	25%	44	24%	
Missing	41	4%	33	4%	8	4%	
**Single gestation**	935	98%	753	98%	182	98%	0.6883
**Multiple gestation**	19	2%	16	2%	3	2%	
**Current smoker**	39	4%	33	4%	6	3%	0.5025
Missing	13	1%	12	2%	1	1%	
**Current alcohol user**	362	38%	305	40%	57	31%	0.0856
Missing	5	1%	4	1%	1	1%	
**Gestational diabetes**	68	7%	61	8%	7	4%	0.0489
**Gestational hypertension**	105	11%	82	11%	23	12%	0.4900
**Psychosocial stressors**	398	42%	316	41%	82	44%	0.4072
Missing	364	38%	295	38%	69	37%	
**COVID‐19 vaccination status prior to pregnancy outcome** ^**d**^							
Not vaccinated	739	77%	576	75%	163	88%	0.0003
Partially vaccinated	33	3%	32	4%	1	1%	
Fully vaccinated	182	19%	161	21%	21	11%	

^a^ SARS‐CoV‐2 infection was defined as ≥1 self‐reported laboratory‐confirmed diagnosis of COVID‐19 during pregnancy before study enrollment, RT‐PCR‐confirmed infection detected by study surveillance, or serologic evidence of SARS‐CoV‐2 infection during the study.

^b^ *p* values were calculated based on a Chi‐square test unless a cell frequency <5, in which case a Fisher exact test was used.

^c^ Based‐on questionnaires that asked about the following medical conditions: asthma, chronic lung disease other than asthma, chronic metabolic diseases such as diabetes mellitus types 1 and 2 and thyroid disease, blood disorders such as thalassemia and sickle cell disease, hypertension, cardiovascular disease, bladder/kidney disease, liver disease, immunocompromising conditions, neurologic/neuromuscular disease, and rheumatologic conditions.

^d^ Includes COVID‐19 vaccination status before enrollment and during study follow‐up.

Of the 954 participants in this analysis, 123 (13%) had preterm live births (GA at delivery 23–36 weeks), including 97/769 (13%) participants without SARS‐CoV‐2 infection during pregnancy and 26/185 (14%) participants with SARS‐CoV‐2 infection. Of the 905 participants with known neonatal outcomes, there were two neonatal deaths that occurred at 0–28 days of life. The adjusted hazard ratio for preterm live birth in the Cox regression model comparing participants with versus without prenatal SARS‐CoV‐2 infection was 1.28 (95% CI 0.82–1.99, *p* = 0.28) (Table 2). The adjusted relative risk from the analogous Poisson regression model was 1.10 (95% CI 0.71–1.72, *p* = 0.67).

**TABLE 2:** Crude and adjusted Hazard ratios of preterm live birth among pregnant individuals with versus without prenatal SARS‐CoV‐2 infection, *N* = 954.

	SARS‐CoV‐2 negative	SARS‐CoV‐2 positive	Unadjusted hazard ratio (95% CI)	*p* value	Adjusted hazard ratio (95% CI)	*p* value
Preterm live birth	97/769 (13%)	26/185 (14%)	1.29 (0.84–1.99)	0.259	1.28 (0.82–1.99)	0.283

*Note*: Adjusted Hazard Ratio estimate for preterm live birth was derived from a Cox proportional hazards model after adjusting for site, age group (18–24, 25–34, and 35–50 years), highest educational level, any chronic conditions, gestational diabetes, gestational hypertension, current smoking status, and multiple gestation.

## 4 DISCUSSION

Using data from a prospective cohort study of >900 pregnant persons followed during early epidemic waves of SARS‐CoV‐2 circulation in the United States, we evaluated the association between largely mild or asymptomatic SARS‐CoV‐2 infection during pregnancy and preterm live birth. Almost one in five persons in this analysis had evidence of SARS‐CoV‐2 infection during pregnancy, and infection incidence was likely higher given that some persons with serologic evidence of infection at cohort enrollment were excluded from the analysis. We found no significant association between preterm birth among pregnant persons with prenatal SARS‐CoV‐2 infection compared with those without infection.

Some previous studies have found a significant association between SARS‐CoV‐2 infection and preterm birth (adjusted relative risk or odds ratio range: 1.2–1.8).^2^, ^6^, ^7^ Infections in our analysis were largely mild or asymptomatic and detected through systematic study testing, whereas these prior studies either focused on severe infections requiring hospitalization and/or infection status determined by clinical testing^8^ that may be influenced by testing biases.^9^ More severe SARS‐CoV‐2 infections may be associated with more robust inflammatory responses that may adversely affect the placenta^10^ and intrauterine environment and predispose to preterm birth. To prevent severe SARS‐CoV‐2 infections that may be associated with additional complications, COVID‐19 vaccination for pregnant persons remains important and is recommended by CDC^11^ and the American Society of Obstetrics and Gynecology.^12^

This analysis has several limitations. First, the sample size was small compared with some surveillance and registry studies, and the study was underpowered to detect an effect size unless it was relatively large. Despite this limitation, this study includes prospectively collected data from a cohort followed with rigorous systematic surveillance for SARS‐CoV‐2 infections and pregnancy outcomes that may contribute to meta‐analyses examining the association between SARS‐CoV‐2 infections and pregnancy outcomes. Second, this study was conducted before the emergence of Omicron variant viruses, and respiratory samples that were positive for SARS‐CoV‐2 were not sequenced to identify virus variants. A previous study suggested that prenatal SARS‐CoV‐2 infection effects on perinatal outcomes may vary by variant type^8^ underscoring the need to assess potential effects on birth outcomes as SARS‐CoV‐2 continues to circulate.

## AUTHOR CONTRIBUTIONS

**Sarita Mohanty:** Formal analysis; visualization; writing—original draft. **Alan Tita:** Conceptualization; investigation; project administration; supervision; writing—review and editing. **Michael Varner:** Conceptualization; investigation; project administration; supervision; writing—review and editing. **Melissa S Stockwell:** Conceptualization; investigation; project administration; supervision; writing—review and editing. **Gabriella Newes‐Adeyi:** Conceptualization; data curation; project administration; supervision; writing—review and editing. **Ashley N. Battarbee:** Conceptualization; investigation; project administration; supervision; writing—review and editing. **Lawrence Reichle:** Data curation; validation; writing—review and editing. **Tyler Morrill:** Data curation; validation; writing—review and editing. **Michael Daugherty:** Data curation; validation; writing—review and editing. **Mirella Mourad:** Investigation; writing—review and editing. **Raul A. Silverio Francisco:** Investigation; writing—review and editing. **Kate Woodworth:** Writing—review and editing. **Kristina Wielgosz:** Data curation; project administration; writing—review and editing. **Romeo Regi Galang:** Investigation; writing—review and editing. **Pete Maniatis:** Investigation; writing—review and editing. **Vera Semenova:** Investigation; writing—review and editing. **Fatimah S. Dawood:** Conceptualization; data curation; funding acquisition; methodology; project administration; supervision; validation; writing—original draft; writing—review and editing.

## CONFLICT OF INTEREST STATEMENT

The authors have no conflicts of interest to declare.

### PEER REVIEW

The peer review history for this article is available at https://www.webofscience.com/api/gateway/wos/peer-review/10.1111/irv.13192.

## DISCLAIMERS

The findings and conclusions in this report are those of the authors and do not necessarily represent the official position of the Centers for Disease Control and Prevention. Any references to specific commercial products are for identification purposes only and do not constitute an endorsement by CDC.

## Supporting information


**Figure S1.** Participant Enrollment and Analytic Populations.

## Data Availability

Data are available upon request to the senior author at hgj0@cdc.gov. Researchers interested in collaborations should contact the senior author. We welcome proposals to use these data from potential collaborators or from researchers interested in investigating specific questions independently.
