# Supplementary figures and images for: Association between SARS‐CoV‐2 infections during pregnancy and preterm live birth
Source: Influenza Other Respir Viruses. 2023 Sep 21;17(9):e13192. doi: 10.1111/irv.13192 (PMC10511836; doi:10.1111/irv.13192)

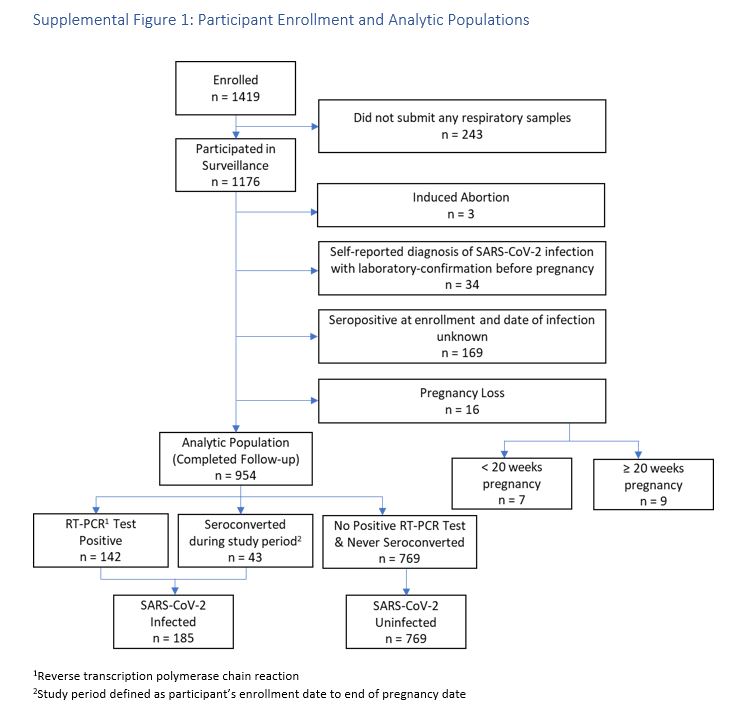

Supplement: Supplementary file 1 — Figure S1. Participant Enrollment and Analytic Populations. [file IRV-17-e13192-s001.jpg]
